# The contribution of plant microbiota to economy growth

**DOI:** 10.1111/1751-7915.13290

**Published:** 2018-06-21

**Authors:** Angela Sessitsch, Günter Brader, Nikolaus Pfaffenbichler, Doris Gusenbauer, Birgit Mitter

**Affiliations:** ^1^ Bioresources Unit AIT Austrian Institute of Technology GmbH Konrad‐Lorenz Straße 24 3430 Tulln Austria

## Introduction

Plant microbiota has been explored in the last decades, particularly those colonizing the rhizosphere, but the awareness of their diversity and relevance has exploded in the last few years. Based on the recent discoveries made with the human microbiome, it has been recognized that also plants host highly diverse microorganisms, which have been suggested to provide an accessory genome and reservoir of important functions to that of the host plant itself contributing to important plant traits. At the same time, agricultural production has to face severe challenges due to the demographic development and climate change with extreme weather events and emerging pathogens. Furthermore, our society demands more sustainable production systems, a number of chemicals (e.g. pesticides) will be taken from the market in the coming years and several countries do not support the use of genetic modification to improve crop traits. All these factors have led to an increasing awareness of the functions mediated by plant microbiota by academia as well as by the industry. Nevertheless, there are still a number of obstacles to face in the application of plant microbiota, and we are just at the beginning to realize their full potential contributing to economic growth and sustainable development.

## Plant microbiota for sustainable intensification of agricultural production

**Figure 1:**
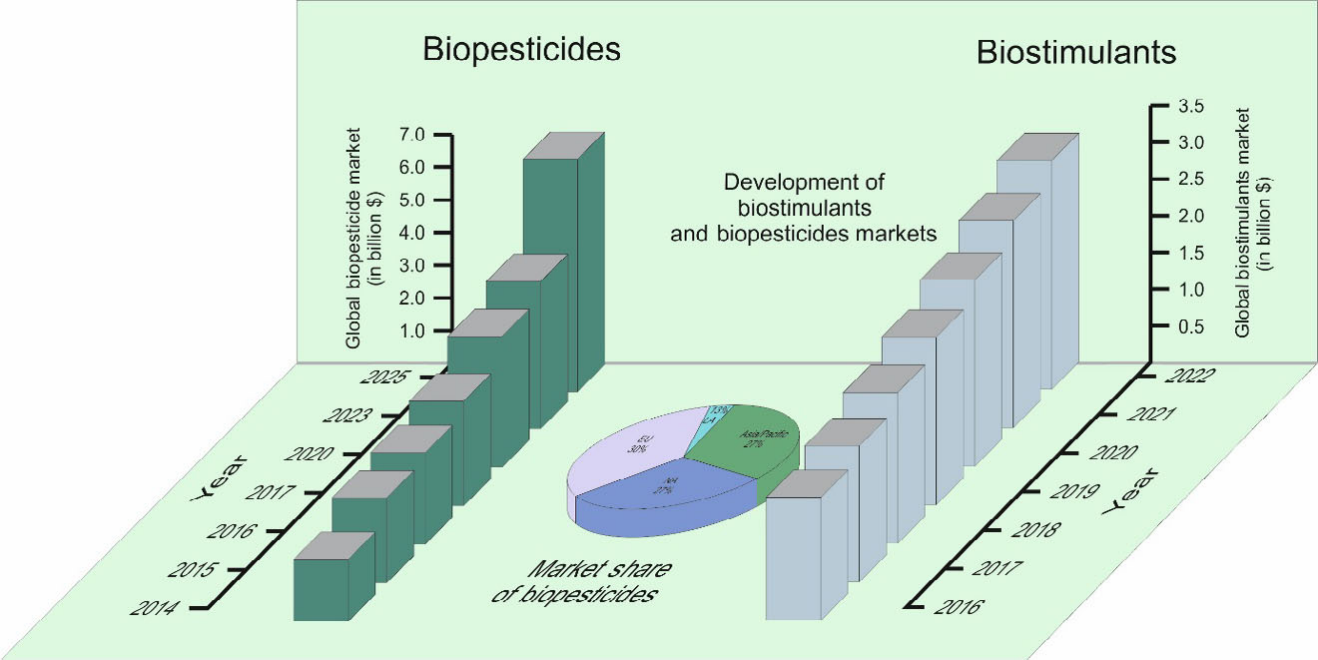
Estimated global market growth of Biopesticides and Biostimulants (in billion $). The biopesticide market was valued at $ 1.8 billion in 2014 with growth prospects of up to $ 7 billion in 2025. The biopesticide market is distributed as follows: 30% European Union EU, 27% North America NA, 27% Asia‐Pacific and 13% Latin America LA (Biopesticides 2016, Agrow Market Report). The Biostimulants market growth was valued at $ 1,68 billion in 2016 with growth potential of up to $ 3.12 billion in 2022 (Biostimulants 2017, Agrow Market Report).

The global population is constantly rising and expected to reach 9.8 billion in 2050 and 11.2 in 2100 (https://www.un.org/development/desa/en/news/population/world-population-prospects-2017.html). At the same time, less land will be available for crop production implying that agricultural management and technologies have to be applied allowing intensification of production. This will be particularly essential for regions with high population densities such as in East and South‐east Asia or regions facing extreme environmental conditions such as in sub‐Saharan Africa. However, global food security is greatly challenged by emerging pathogens and climate change facing rising temperatures, extreme weather events and long periods of drought. There are a number of technologies available or under development such as precision agriculture or also genetically engineered plants being able to cope with various stresses. Nevertheless, the latter are not well accepted in many parts of the world. Currently, there are great expectations in the application of microbial inoculants as promising results have been reported and so far this approach has been hardly applied in crop production with the exception of N_2_‐fixing rhizobial inoculants for legume production. In many parts of the world, a number of start‐up companies have emerged exploring microbial inoculants and sophisticated ways to make use of the plant beneficial activities of microorganisms. The major drivers for economic development of this sector are the global demographic development and the increasing yield loss due to abiotic stress (e.g. drought), the lack of chemistry and active ingredients with new modes of action, resistance development of pathogens and pests against chemical treatments, the pressure from society and regulators for reduced pesticides on food and the environment, the adoption of integrated pest management in Europe and other countries as well as arising opportunities in the organic food sector (Biopesticides 2016, Agrow Market Report; Biostimulants 2017, Agrow Market Report). The global microbial biopesticide market accounted in 2014 more than 800 million $ (Biopesticides 2016, Agrow Market Report), a more recent analysis reported a global biocontrol market of 2.8 billion $ today to over 11 billion $ in 2025 with about 60% microbial products (Dunham Trimmer 2017). A CAGR (compound annual growth rate) of 17% is expected for the years 2015–2020 (Dunham Trimmer 2017). Similarly, the biostimulants market is constantly increasing with an expected CAGR of 10.9% until 2022 (Biostimulants 2017, Agrow Market Report). Dunham Trimmer (2017) reported outside of India and China currently more than 200 biocontrol and more than 100 biostimulant companies. About 75% of the biocontrol companies have less than 10 million $ annual return while about five biocontrol companies have an annual return of more than 100 million. Generally, all major global seed companies have invested in the field of plant microbiome research and chemical companies increasingly explore new commercial opportunities with developing microbial alternatives to agrochemicals (Fig. 1).

The exploration of plant microbiota can be particularly relevant for small and medium enterprises and those acting in less developed regions. Since 2000, global efforts to combat hunger and malnutrition have advanced significantly. However, some regions such as in Asia and Africa still suffer tremendously from hunger, food insecurity and malnutrition and technologies to increase agricultural productivity are urgently required. There, microbial inoculants can be well produced locally by small companies and applied in small‐scale farms and have the capacity to ameliorate poor soils frequently occurring in such countries. Great progress has been made in South America and best practice example is *Azospirillum*. Members of this genus are well known for their capacity to promote plant growth by providing phytohormones and nutrients. Based on major national funding programs, in which promising *Azospirillum* strains were selected, a flourishing inoculant business has arisen. Microbiologists, agronomists and local industries have meanwhile rigorously tested various inoculants in the field and suitable formulations have been developed. Nowadays, in South America, there are more than 100 commercial products available containing *Azospirillum* strains, most of them are produced in Argentina and Brazil. These products are produced by more than 50 companies and aim at yield increase in maize, wheat and soybean (reviewed by Cassán and Díaz‐Zorita, 2016).

## Soil sanitation and improvement by employing plant microbiota

Securing and improving sanity of soil and water is a central challenge in face of climate change and all its consequences and is an explicit UN development goal. Considering the importance of plant‐associated microbiota for host and ecosystem functioning the exploitation of microbial activity could provide means to achieve this goal on different levels. The application of microbials with plant growth‐promoting or biocontrol activity could at least partly substitute agrochemicals, thereby reducing their release into soil and water and consequently the negative effects on the environment. A proposed management strategy to increase soil health is to apply rhizobacteria to fill vacant niches and thereby prevent pathogen invasion (Chaparro *et al*., 2012). Furthermore, making use of the plant stress resilience effect of many plant‐associated microorganisms could help to save water and natural soils by increasing yield per acre farmland. Apart from preventing contamination, plant microbiota can also be employed in decontamination of polluted soils. Many plant species take up metals from the surrounding soil and store them in their tissues. In a process called phytoextraction such accumulator plants are planted on contaminated sites and the metal‐containing biomass is harvested and the soil recovers and is made available for agricultural use (Moosavi and Seghatoleslami, 2013; Kidd *et al*., 2015). The activity of plant microbiota can further enhance the efficiency of phytoextraction, as many bacteria mobilize metals in soil and so facilitate the uptake by plants. Others promote leave growth, which in turn allows incorporation of higher amounts of metals per plant (Kidd *et al*., 2015). These microbe‐assisted processes could also be employed as gentle and less‐invasive alternative to conventional mining, by extraction of valuable metals accumulated in plant tissue (Ghasemi *et al*., 2018). Furthermore, plant microbiota partnerships enable clean‐up of soils and groundwater from different organic pollutants (Afzal *et al*., 2014). Many plant‐associated bacteria possess enzymes for the mineralization of different organic compounds, thereby reducing organic compound accumulation and transpiration in plant tissue and allowing for better plant growth (Afzal *et al*., 2014).

## Implications for human health

The role of plant microbiota in plant health, productivity and ecosystem functioning is well acknowledged (Berg and Smalla, 2009); however, the activities of plant‐associated microorganisms can also affect human health and well‐being. The microbial‐based management strategies for reduced use of agrochemicals or soil and water sanitation mentioned above certainly will have positive effects on human health by reducing the exposure to potentially harmful chemicals and metals. However, the plant microbiota also directly affects humans, as it consists not only of plant beneficial, neutral and plant pathogenic bacteria but comprises also potential human pathogens (Mendes *et al*., 2013), which are taken up by the human body through consumption of raw plants such as vegetables and fruits (van Overbeek *et al*., 2014). Furthermore, it was assumed that plant microbiota is interconnected with those of humans also via air, soil, animals and indoor environments (Berg *et al*., 2014). Consequently, strategies to ensure healthy and balanced plant microbiota, such as prebiotics for plants, could play an important role in preventing disease outbreaks in humans (Berg *et al*., 2014).

## Plant microbiota as a huge reservoir of novel bioactivities

Microbial enzymes and metabolites have been long essential products for a number of industrial processes. More than half of the commercial enzymes are fungal or bacterial origin, while a smaller part is derived from animals or plants (Borrelli and Trono, 2015). The market for industrial enzymes is projected to reach more than 6 Billion USD by 2022 with a projected annual growth rate of close to 6%. The rising demand for alternatives to energy‐intensive chemical technologies led to expectations for further increasing interests in enzymes and biocatalysis (Prakash *et al*., 2013; Markets and Markets 2018). In addition, microbial (secondary) metabolites have been a rich source of natural products commercialized in animal and human medicine and health, food and chemical industry and in plant crop protection. In 2013, app. 40% of the new chemical entities approved by the US Food and Drug Administration are either natural products, mimics or derivatives, and approximately 75% of antitumor and anti‐infective agents are derived from natural products, largely of bacterial origin (Katz and Baltz, 2016). The total microbial products (diagnosis and treatment) market is expected to reach more than 250 billion USD in 2023, with a 40% fraction in the pharmaceutical industry and an annual growth rate of more than 8% from 2017 on (Market Research Future, 2018). In agriculture, metabolites of microbial origin are less prominent in use, but different metabolites such as the insecticide spinosyn from *Saccharopolyspora* (Dayan *et al*., 2009) and biosurfactants from various *Bacillus* and *Pseudomonas* species already provide or are expected to provide products with high potential in application in agriculture (Sachdev and Cameotra, 2017). Despite this already large contribution of microbial products to different industries, the overall potential of microorganisms is far from exhaustively exploited as the majority of bacteria (> 90%) are uncultivable with conventional techniques and only a small sub‐fraction of metabolic clusters are active in most cultivated microorganisms. This ‘biosynthetic dark matter’ can now be accessed by high throughput sequencing techniques and have high potential to provide a number of novel lead structures and compounds (Katz and Baltz, 2016). Due to adaptation to different host niches, differences in the chemical composition of plant hosts and the intense interaction with other microorganisms on and in plants, the plant microbiome provides a hotspot of specific and diverse metabolic and enzymatic potential of microorganisms (Aleti *et al*., 2017; Hassani *et al*., 2018). Indeed, sequence comparison within Firmicutes has revealed an enrichment of genomic clusters responsible for secondary metabolite production particularly in those strains, which have been found in association with plants (Aleti *et al*., 2015). As the high number of different very specific functions (including utilization of diverse metabolic products, degradation of toxins and signalling compounds of microorganisms and plants, adaptation to abiotic stress conditions) are needed to allow survival of microorganisms in plant associations, these adaptations are expected to be reflected in high diversity of metabolites and enzymes produced by plant‐associated organisms (Brader *et al*., 2017).

## The necessity to establish microbial gene banks

It is widely recognized that maintaining the genetic diversity of plants and animals is crucial for agriculture and food production. In 2016, 4.7 million samples of seeds and other plant genetic material for food and agriculture have been preserved in 602 gene banks throughout 82 countries and 14 regional and international centers (https://sustainabledevelopment.un.org/sdg2). It is similarly important to increase awareness on the importance of microorganisms being tremendously important for our life and well‐being as well as for maintaining environmental functions. Soils are central components of healthy and functional ecosystems and in particular soil organisms play a key role for soil functioning. Considering the importance of mostly soil‐derived microorganisms and microbial communities for plant growth, health and stress resilience, the commercial impact of soil‐borne and plant‐associated microbiota is immense. Recently, Manter *et al*. (2017) claimed that the establishment of Living Soil Repositories is needed and that such repositories represent an investment in our future. Living Soil Repositories could preserve genetic diversity and could serve as a baseline and future tool to monitor changes in community structure and functioning. Such a repository would also open commercial opportunities to make use of microbiota for agricultural needs (Manter *et al*., 2017). Furthermore, Berg and Raaijmakers (2018) pointed out that the centralized seed production and the global trade of seeds can lead to more homogenous plant microbiomes, potentially loosing important microbial players. The authors propose that international seed banks should also consider and maintain seed‐associated microbial diversity to save microorganisms, which are important for securing global food production as well as economic viability.

## Final remarks

In the last decade, the importance of plant microbiota has gained increasing awareness, which is evident from the number of publications and ongoing projects in this field. Also, the industry sector is extremely interested in microbial applications to improve crop production. Nowadays more than 300 companies comprising big international players in this sector as well as small and medium enterprises participate in the Annual Biocontrol Industry Meeting (http://www.abim.ch/home.html) organized every year by the International Organization of Biocontrol Manufacturers in Switzerland. This international meeting has been in place for many years but participant numbers have started to explode recently demonstrating the tremendous interest by the industry. This interest is driven by major challenges such as the demographic development or climate change we nowadays face in crop production. Due to the high application potential, major investments in this sector are made and expectations are high. It will be important to manage these expectations appropriately to avoid disappointments and the premature termination of promising R&D strategies. Apart from applications in the crop production sector plant microbiota host an extremely diversity of microorganisms and functional activities, which might be equally relevant for applications in other fields such as soil sanitation or medical treatments. Overall, the public and policy sector has to be aware that plant microbiota is essential for the health and growth and plants as well as for the functioning of terrestrial ecosystems. Plant microbiota is not only an important component of biodiversity, but also have the potential to further contribute to the economic development in developed as well as less developed parts of the world.

## Conflict of interest

None declared.
